# Neuralgia due to iliohypogastric nerve injury after inguinal hernioplasty: a case report

**DOI:** 10.1186/s12893-018-0391-6

**Published:** 2018-08-16

**Authors:** Kamleshsingh Shadhu, Dadhija Ramlagun, Simeng Chen, Lijia Liu

**Affiliations:** Department of General Surgery, Jiangsu Province Hospital and The First Affiliated Hospital of Nanjing Medical University, Guangzhou road, 300, Gulou district, Nanjing, Jiangsu 210029 People’s Republic of China

**Keywords:** Neuralgia, Iliohypogastric nerve entrapment, Sensory disturbances, Partial neurectomy

## Abstract

**Background:**

Neuralgia due to iliohypogastric nerve entrapment from sutures and mesh after inguinal hernioplasty is a rare entity in clinic. Its’ awareness and management remain a clinical challenge.

**Case presentation:**

We report a case of 54-year-old male who presented with post-operative pain after 1 month and sensory disturbances of the right lower limb. He underwent partial neurectomy and during the surgery it was found that there was injury to iliohypogastric nerve due to entrapment from sutures and mesh.

**Conclusion:**

We hope to strengthen awareness about the importance of the identification of iliohypogastric nerve during inguinal hernioplasty.

## Background

Postoperative pain after surgery of the right lower abdomen and inguinal region is an underestimated problem [1]. About 11% of all patients undergoing an inguinal hernioplasty will develop discomfort [2, 3]. The main cause of inguinal pain is injury or irritation to the following nerves: ilioinguinal, iliohypogastric and genital branch of genitofemoral nerve [4]. Mechanical or thermal injury during surgical dissection and repair, nerve entrapment from sutures, staples, mesh, and adhesions or injuries related to the inflammatory response to prosthetic mesh material, are factors contributing to nerve injury [5, 6]. Nerve damage incurred during surgery can cause acute pain just after the surgery. Sensory disturbances are frequently seen in these patients and it appears to be the most common aetiology [7]. The distribution area of the affected nerve is usually painful. The ilioinguinal nerve is known as to be the most common neural pain generator. It is followed by the genitofemoral nerve, and iliohypogastric nerve. So far, several treatment regimens have been proposed, although high level evidence is scarce [8]. We report a case of a patient with sensory disturbances with acute, unbearable and disabling pain in the right lower limb for 1 month after a right inguinal hernioplasty.

## Case presentation

**Fig. 1:**
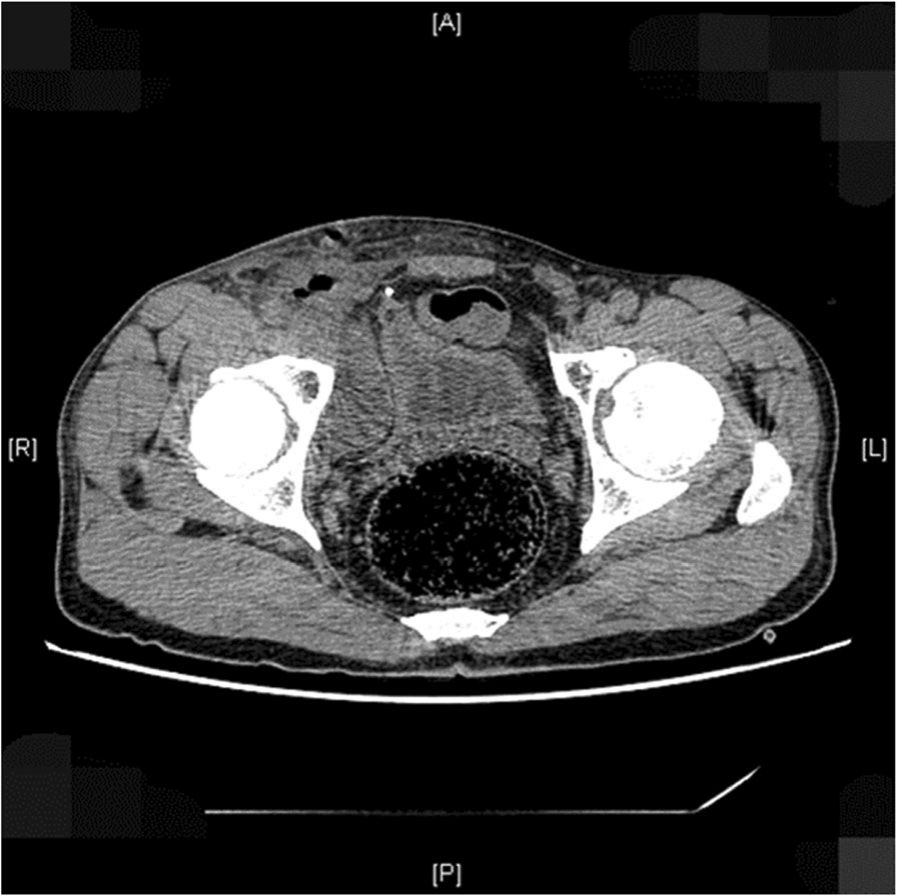
CT scan of the patient

**Fig. 2:**
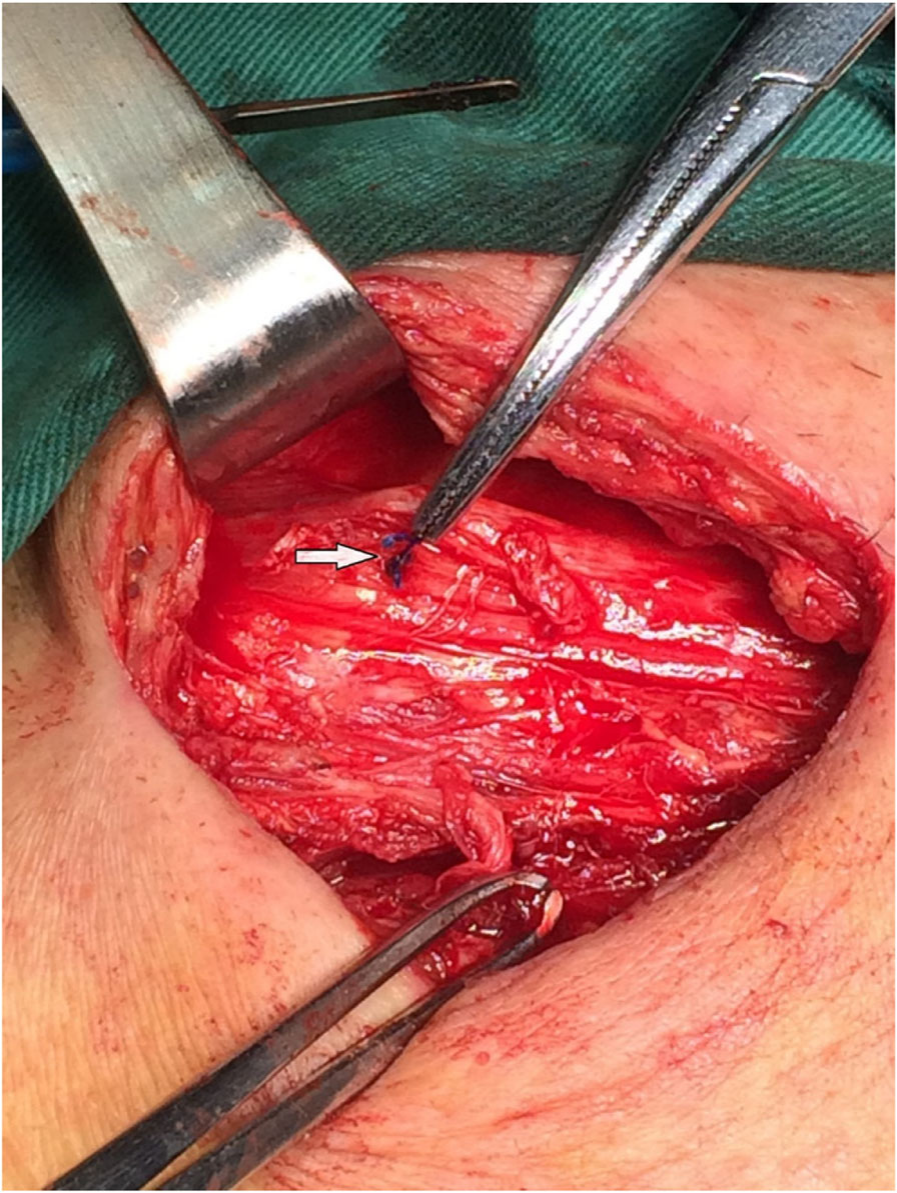
White arrow showing the iliohypogastric nerve entrapment from sutures and the ‘Kugel’ mesh, found during the surgery

A 54-year-old male patient presented with post-operative pain for 1 month. He underwent right inguinal hernioplasty 1 month ago at a different hospital. The patient complained about pain, of score 9 (on a scale of 1–10; 1 being normal 10 being most severe), around the right medial thigh and the pain intensified when he stands or walks for a long time and complains about abnormal walking posture. This situation was accompanied by lateral hip joint pain: the hip joint was unable to adduct and sometimes the pain can be felt at the knee joint if severe enough. It was also accompanied by pain during micturition which however, was relieved after rest. The CT scan showed bilateral hip joint degenerative changes, oedema at the region of the right inguinal hernioplasty and encapsulated effusion at the right pelvic cavity *(*Fig. 1*)*. He was diagnosed with neuralgia after right inguinal hernioplasty. When he was admitted to our hospital, local anaesthetics were used at two points: one just above the right pubic tubercle and the other 2 fingers above the right inguinal ligament and medial to the right anterior superior iliac spine. The pain was alleviated temporarily for just 2 hours. Conservative regimen which included pain killer Tramadol failed. He underwent partial neurectomy under general anaesthesia based on his right to autonomy. During the surgery, the spermatic cord was freed, and the mesh was visible. Modified Kugel mesh procedure was carried out in the previous surgery during which 4 sutures over the transversalis fascia were stitched. The mesh had adhesion with the surrounding tissues, with unclear boundaries. The mesh was exposed and right iliohypogastric nerve was observed on the superficial surface of the mesh. The right iliohypogastric injury was due to entrapment from 3 sutures and the ‘Kugel’ mesh *(*Fig. 2*)*. The genitofemoral nerve was explored for any abnormality. The nerves were identified based on their courses. The 4 non-absorbable sutures were all removed since the mesh has been integrated to the surrounding tissues. Tailored neurectomy of the iliohypogastric nerve was carried out whereby 3.5 cm of nerve length was resected. The proximal ends were cauterized. On POD 1 the patient stated that the pain was relieved and restored to ambulation and he had no pain during micturition either. He was discharged on POD 3. There has been no further complaints or abnormalities from this patient during follow-ups.

## Discussion & Conclusion

The surgical approach for neurectomy is still a controversial issue. The traditional anterior approach to the ilioinguinal and iliohypogastric nerves [9], has been reported to have success rates between 30 and 100% [10, 11]. Surgery as an option for treating nerve-associated pain was described by Stulz and Pfeiffer, in 1982 [12]. Cicatricotomy to treat inguinal neuralgia after inguinal herniotomy, appendicectomy, and gynecological operations, was performed by them. Surgical neurectomy is the optimum method of treatment when conservative treatment has failed, and it was postulated that ilioinguinal nerve is the most endangered after hernia surgeries [5, 10, 11, 13]. This shows that our case is even rarer due to the involvement of iliohypogastric nerve. Moreover, Vuilleumier used an open approach to perform a radical ilioinguinal nerve and iliohypogastric nerve neurectomy without routinely dissecting the genitofemoral nerve, having 95% pain relief in 49 patients [11]. Some authors found beneficial results resecting only the specific nerves that fit the clinical pain syndrome- ‘tailored neurectomy’ [13]. In addition, the use of cross-sectional computed tomography (CT) has been advocated to exclude recurrence or meshoma and investigate other differential diagnoses of chronic postoperative inguinal pain [14]. This explains the use of CT scan as imaging modality in our case.

Up to now there has been no specific guidelines about the treatment of this clinical entity and surgical neurectomy has proved to be the most effective treatment modality.

We, therefore, reported this case to increase awareness among clinicians and stress on the importance of the identification of iliohypogastric nerve in open hernioplasty. However, our limitation is that it is a single case experience and conclusion can only be drawn based on our experience.

## Abbreviations

CT: Computed Tomography
POD: Post-Operative day
